# A semi-automatic and quantitative method to evaluate behavioral photosensitivity in animals based on the optomotor response (OMR)

**DOI:** 10.1242/bio.033175

**Published:** 2018-06-15

**Authors:** Megumi Matsuo, Yoriko Ando, Yasuhiro Kamei, Shoji Fukamachi

**Affiliations:** 1 Department of Chemical and Biological Sciences, Japan Women's University, Tokyo 112-8681, Japan; 2 Spectrography and Bioimaging Facility, National Institute for Basic Biology, Aichi 444-8585, Japan; 3 Department of Basic Biology, School of Life Science, The Graduate University for Advanced Studies (SOKENDAI), Aichi 444-8585, Japan

**Keywords:** Spectral sensitivity, Optomotor response (OMR), Medaka, Long-wavelength sensitive (LWS), Red colorblindness

## Abstract

The optomotor response (OMR) is a locomotor behavior of animals that is induced by moving repetitive visual stimuli. This characteristic helps animals particularly when stabilizing and maintaining position in schools and herds. Here, we developed a simple but sensitive method for quantifying the OMR using medaka (*Oryzias latipes*) as a model. This method, which simply requires video-recorded behavior, free tracking software, and a generic spreadsheet program, enables the evaluation of spectral sensitivity by researchers with little knowledge about the behavioral characteristics of the test animal or of the OMR. Based on a manual method, we reported previously that wild-type and red-colorblind medaka exhibited an OMR up to λ=830 and 740 nm, respectively. However, the present method, which quantifies the OMR according to three parameters (starting time, duration, and total distance of swimming) that are calculated based on a series of x–y coordinates of the moving fish, supported that conclusion and further indicated that both strains perceive light at even longer wavelengths. This low-cost, quantitative, and semi-automatic method would widen the opportunities to unveil behavioral photosensitivity in animals of interest.

This article has an associated First Person interview with the first author of the paper.

## Abstract (precis)

**Summary:** A semi-automatic analytical method for the assessment of the optomotor response was developed. This procedure was animal friendly and enabled the quantitative evaluation of fish optic behavior.

## INTRODUCTION

The eyes of humans perceive light using four types of opsins: long-wavelength sensitive (LWS or OPN1LW), mid-wavelength sensitive (MWS or OPN1MW, which is evolutionarily paralogous to LWS), short-wavelength sensitive 1 (SWS1 or OPN1SW), and rhodopsin 1 (RH1 or RHO). Fish generally have more copies of the *opsin* genes. For example, the genome of zebrafish (*Danio rerio*) contains a single violet (*SWS1*), a single blue (*SWS2*), four green [*rhodopsin 2* (*RH2*)*-1*, *RH2-2*, *RH2-3*, and *RH2-4*], and two red (*LWS-1* and *LWS-2*) *cone-opsin* genes, in addition to two *rod-opsin* genes (*RH1* and *RH1-2*) (Chinen et al., 2003; Morrow et al., 2011). The peaks of the absorption spectra (λ_max_) of these proteins are 355 nm (SWS1), 416 nm (SWS2), 467 nm (RH2-1), 476 nm (RH2-2), 488 nm (RH2-3), 505 nm (RH2-4), 548 nm (LWS-2), 558 nm (LWS-1), 500 nm (RH1-2), and 501 nm (RH1). Nile tilapia (*Oreochromis niloticus*) carry seven *cone-opsin* genes: *SWS1*, *SWS2b*, *SWS2a*, *Rh2b*, *Rh2a beta*, *Rh2a alpha*, and *LWS*, with corresponding λ_max_ values of 360, 423, 456, 472, 518, 528, and 561 nm, respectively (Spady et al., 2006). In medaka (*Oryzias latipes* and *Oryzias sakaizumii*), eight *cone-opsin* genes and one *rod-opsin* gene have been identified, with corresponding λ_max_ values of 356 nm (SWS1), 405 nm (SWS2b), 439 nm (SWS2a), 452 nm (RH2a), 492 nm (RH2c), 516 nm (RH2b), 561 nm (LWSa), and 562 nm (LWSb) (Matsumoto et al., 2006). Although these molecular absorbances are informative, fish can respond physiologically and behaviorally to light out of these ranges.

The optomotor response (OMR) is a visually induced locomotor behavior of animals that occurs when the animal is pursuing a moving repetitive stimulus pattern. This ability affords synchronized and coordinated movements to fish, such as flocking and schooling behaviors (Imada et al., 2010; Wark et al., 2011). In most studies, the stimulus pattern that is used to induce the OMR consists of a vertical black and white striped pattern that is rotated around the test animal (Rahmann et al., 1979). The OMR is displayed by any animal that requires orientation or stabilization of its position. Therefore, it has been a very useful tool for elucidating eye function in many aquatic animals, such as zebrafish, sticklebacks, goldfish, crayfish, and tadpoles (Cronly-Dillon and Muntz, 1965; Dong et al., 2009; Glantz, 2001; Maaswinkel and Li, 2003; Rick et al., 2011). The OMR has also been observed in land animals, such as *Drosophila*, honeybees, stick insects, and rodents (Durr, 2005; Seelig et al., 2010; Stojcev et al., 2011). These studies used the OMR to investigate photosensitivity, color perception, or the mechanisms of leg coordination during walking.

The OMR has also been used efficiently in medaka to investigate spectral sensitivity (Hasegawa, 1998), athletic capability (Kawasaki et al., 2008), and cohesive movement (Imada et al., 2010). These small fish school in nature, indicating their preference to associate with moving objects, such as vertical stripes; i.e. this fish is sensitive to the visual stimulus and stabilizes its position among conspecifics. Thus, medaka could be a good model for analyzing physiological or behavioral characteristics using the OMR. In fact, we also analyzed the OMR to demonstrate reduced spectral sensitivity in red-colorblind medaka, which were established by introducing double-frameshift mutations into the *LWSa* and *LWSb* genes (Homma et al., 2017). Using the Okazaki Large Spectrograph (OLS) as a source of monochromatic light (Watanabe et al., 1982), we reported that the longest wavelengths that could be perceived by the wild-type and *lws^–^* medaka were 830 and 740 nm, respectively.

In medaka, the OMR was so apparent that it initially seemed we could even discriminate wild-type from *lws^–^* fish by observing their behavior at λ=760 nm; i.e. among 77 fish with an unknown genotype, all 48 fish that were judged by us as being OMR positive were in fact wild-type animals (i.e. subsequent genotyping of the *LWS* loci revealed that they were homozygous or heterozygous for the wild-type alleles). However, the remaining 29 fish, which were classified by us as OMR-negative animals, included 19 *lws^–^* and ten wild-type fish. Thus, our assessment did not include any type I errors (false-positive results) but included type II errors (false-negative results). Medaka sometimes showed little interest in the rotating stripes [e.g. pecking something at the bottom, associating with the tank wall (a mirror image of itself?), or just swimming around restlessly; see also the Results section]. In such cases, we stopped and repeated the OMR test after several minutes. For the ten wild-type fish, however, we likely overlooked such indifference to the rotating stripes, did not repeat the test, and misclassified the animal as being OMR negative.

The manual assessment of the OMR requires rather specialized skills of behavioral observation; i.e. the ability to discriminate fish that intentionally do not from those that physiologically cannot exhibit an OMR is essential. Fish often try to escape from, and are distracted by the stimulus, which would be deemed as a negative response. In addition, the OMR is not an all-or-none response and may be quantified using appropriate parameters, which would identify a gradual attenuation of photosensitivity at the visible–invisible boundary. Based on these standpoints, we established a mathematical and quantitative method to measure the OMR more accurately and reanalyzed the spectral sensitivities of wild-type and *lws^–^* medaka.

## RESULTS

### Tracking using UMATracker and its efficiency

Using five and six adult fish as the control and mutant groups, respectively, we individually performed the OMR test using the OLS as a light source (the O–O test) and recorded the behavior of the animals. Because the control and mutant fish had exhibited an OMR up to 840 and 730 nm, respectively, in our previous study (Homma et al., 2017), we chose wavelengths of 720 nm, 750 nm, and every 10 nm between 800 nm and 850 nm for the control and every 10 nm between 720 nm and 760 nm, 800 nm, and 850 nm for the mutant, to focus on behaviors at the visible–invisible boundary. Thus, the behaviors could be compared directly between the strains at λ=720, 750, 800, and 850 nm. After the series of the O–O tests (five controls under eight wavelengths+six mutants under seven wavelengths), we obtained a total of 82 movies, which were analyzed using a free tracking software, the UMATracker program (http://ymnk13.github.io/UMATracker/).

**Fig. 1.**
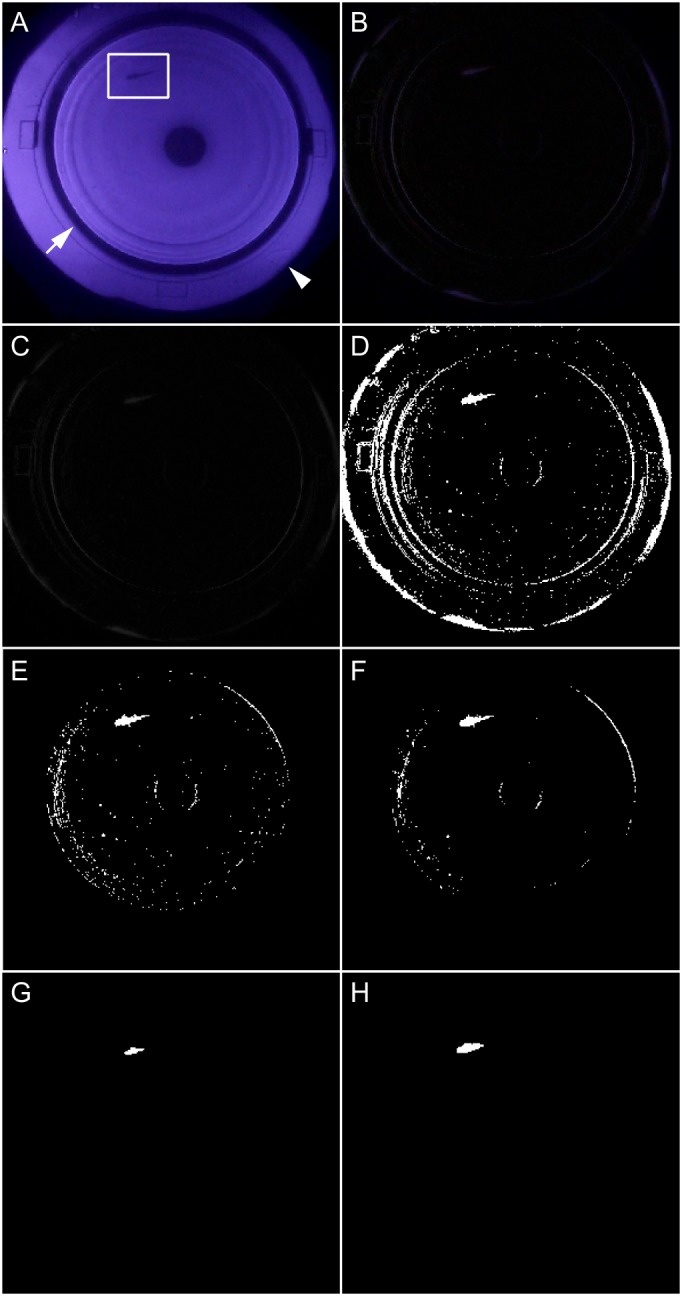
**Generation of binarized movies of medaka behavior during the O–O test using the UMATracker program.** (A) An original image recorded by the video camera. The rotating stripes were at the edge of a purple circle (arrowhead), and medaka swam within the inner circle (i.e. the cylindrical tank; arrow). The dark circle at the center is the 50 ml tube that was used to prevent shortcut swimming during the OMR. The irradiated light appeared as red to the human eye, but was recorded as purple by the IR-recording video camera. (B) Background subtraction. All objects that did not move from the beginning to the end of the movie were regarded as background and subtracted from each frame, which retained the medaka, rotating stripes, and noise items (e.g. feces at the bottom of the tank or reflection at the surface). (C) Conversion to gray scale. The RGB image was converted to gray scale prior to binarization. (D) Binarization. By setting an appropriate threshold, only medaka should become white, while other items should become black. In this image, the rotating stripes and light fluctuations remained as noise, which had to be eliminated in the subsequent steps. (E) Definition of the tracking area. The noise from the rotating stripes was excluded. (F) Noise reduction I. The Median filter removed small dots without affecting big dots. (G) Noise reduction II. The Erosion filter reduced the size of all dots. Only the largest dot (i.e. medaka, in most cases) remained after this filter was applied. (H) Noise reduction III. The Dilation filter recovered the volume of the remaining dots. This step was dispensable but helpful in detecting the remaining noise manually. If such remaining noise was detected in this step, the Median and/or Erosion filters were applied again, because any noise may disrupt tracking. The goal of this binarization was to obtain a movie in which a single white dot was moving on a black background.

Because the frame rate of the video camera (A10FHDIR; Kenko, Tokyo, Japan) was 60 fps and each O–O test lasts ∼2 min (see the Materials and Methods section), each movie consisted of ∼7200 frames. All frames were binarized using appropriate filters (Fig. 1) and were used for tracking. During this binarization, we needed to focus on removing noise; thus, in some cases, no object remained for tracking. Accordingly, some frames lacked the x–y coordinate (i.e. position of medaka), which occurred particularly when the medaka swam close to the tank wall. However, even in such cases, the high tracking efficiency (the number of frames with a coordinate / the total number of frames) obtained (98.3%±0.0%; mean±s.e.m.; *n*=82) indicated that the binarizing procedure was appropriate (Table 1).

**Table 1.**
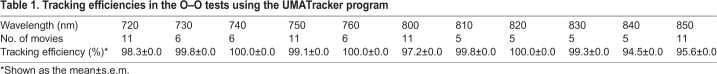
**Tracking efficiencies in the O–O tests** **using the UMATracker program**

### Analyses of the coordinates

**Fig. 2.**
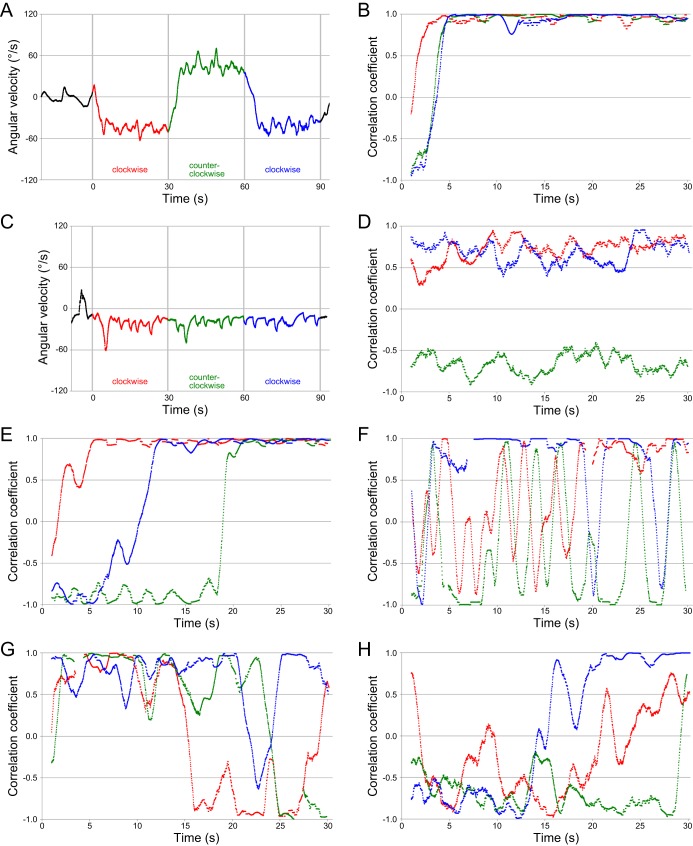
**Examples of changes in angular velocity and correlation coefficient during the O–O test.** The values in the first clockwise, second counterclockwise, and third clockwise stripe rotations are shown in red, green, and blue, respectively. The angular velocity became ±36°/s and the correlation coefficient became +1.0 when fish followed the rotating stripes exactly. (A,B) A typical example of OMR-positive fish (control fish at λ=750 nm). The fish quickly started and stably continued following the stripes in all three rotations. (C,D) A typical example of OMR-negative fish (mutant fish at λ=850 nm). The fish continued swimming slowly in a clockwise direction (without any turning) throughout the 90 s O–O test. (E,F) Examples of fish that did not follow the stripes. Mutant fish at λ=760 nm (E) immediately started the OMR in the first rotation, but not in the second or third rotations. Whether the fish overlooked or intentionally ignored the changes in rotating direction is unknown. Control fish at λ=720 nm (F) restlessly swam back and forth in the first and second rotations, whereas the correlation coefficient became stably high for at least ∼10 s in the third rotation. These quick changes in swimming direction were rarely observed when fish could not follow the stripes (see Fig. 2D). (G,H) Examples of fish with an ambiguous behavior. Whether medaka did not or could not follow the stripes was unclear. A control fish at λ=850 nm (G) largely swam in the rotating direction, but the values fluctuated comparison with those shown in Fig. 2B. A mutant fish at λ=750 nm (H) seemed to start following the stripes at the end of each rotation, but this could be an accidental correlation.

The series of x–y coordinates generated by the UMATracker was analyzed using Excel software (Microsoft). The angular velocity and correlation coefficient between the directions of swimming and the rotation of the stripes were necessary and sufficient to mathematically describe the swimming of medaka (i.e. the movement of the coordinates) in the cylindrical tank with an obstacle at the center (see the Materials and Methods section). When fish exhibited a typical OMR during the O–O test, the angular velocity immediately changed from a negative (clockwise) to a positive (counterclockwise) value, and vice versa whenever the rotation was switched (Fig. 2A). Moreover, the correlation coefficient exceeded 0.9 immediately and was kept constant in every rotation (Fig. 2B). In contrast, when the irradiated light was invisible, and fish could not exhibit an OMR, the switching of stripe rotation had no apparent effect on the angular velocity (Fig. 2C) or on the correlation coefficient (Fig. 2D).

As mentioned in the Introduction, the OMR is not a reflex behavior and medaka sometimes ignored or swam against the rotating stripes (e.g. Fig. 2E,F). In these cases, neither the angular velocity nor the correlation coefficient was an efficient index to detect the OMR, which would cause type II errors (false-negative results), as in the manual observation. Although the changes in correlation coefficient looked rather different between the fish that could not follow the stripes under invisible light (Fig. 2D) and the fish that did not follow the stripes under visible light (Fig. 2F), the difference was often ambiguous at the visible–invisible boundary (e.g. Fig. 2G,H). Because there seemed to be no appropriate index for the discrimination of the former from the latter, we used all data in the following analyses.

### Parameters used to quantify the OMR and spectral sensitivity of the control and mutant medaka

Using the angular velocity and correlation coefficient, we tested three parameters that seemed to be consistently effective, as described below.

**Fig. 3.**
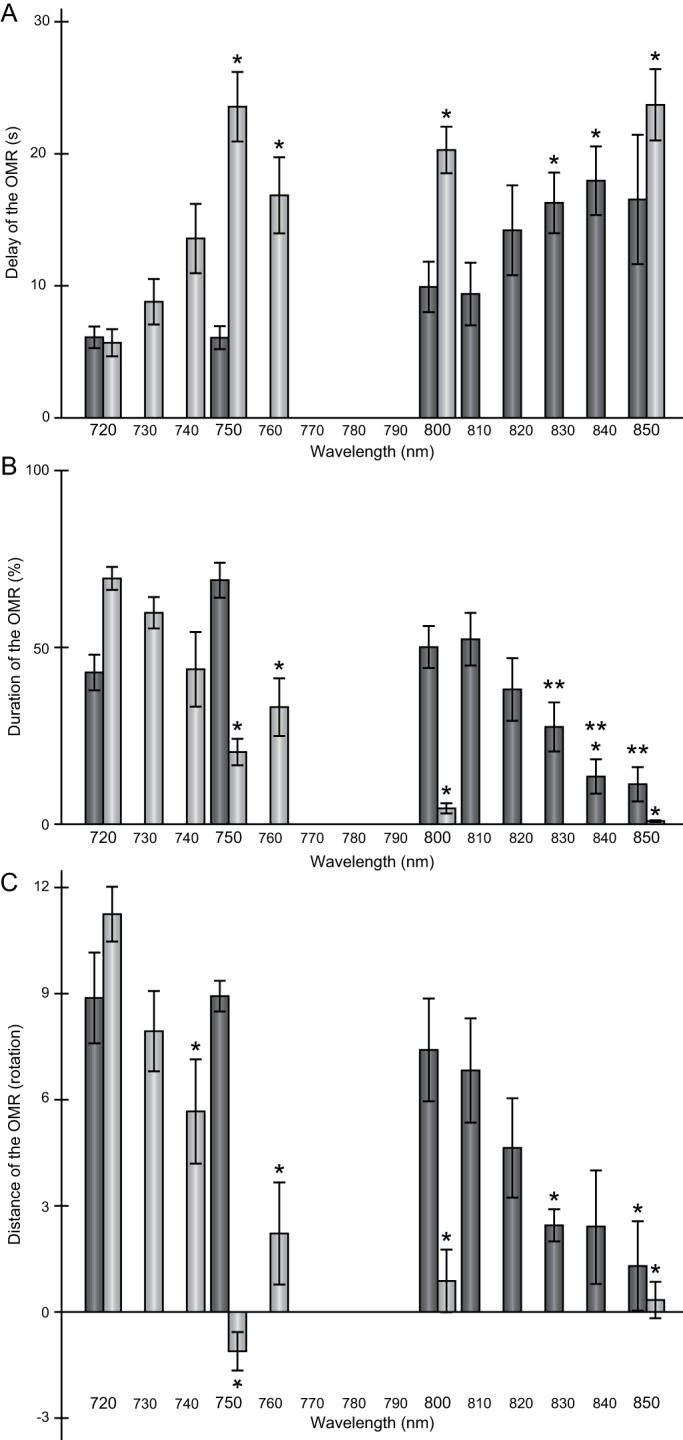
**Parameters used to quantify the OMR in this study.** Dark gray, control fish; light gray, *lws^–^* fish. Means±s.e.m. are shown. Asterisks indicate significant differences compared with the data collected at λ=720 nm, according to Steel's test. (A) Time elapsed (in seconds) until the correlation coefficient reached a value of 0.9 after the start/switching of the stripe rotation (delay of the OMR). The control fish showed a significant delay at λ≥830 nm, whereas the *lws^–^* mutants showed a delay at λ≥750 nm. The delay recorded for the control at λ=850 nm was not significant because of the large variance observed. (B) Duration of the maintenance of the correlation coefficient at a value of 0.9 or more (duration of the OMR). In the control fish, this duration was significantly shorter only at λ=840 nm. However, compared with the duration observed at λ=750 nm (the highest value), the elapsed time was significantly and consistently shorter at λ≥830 nm (double asterisks). At λ=720 nm, four of the five control fish were restless (Fig. 2F, and other data not shown), which resulted in an accidentally short duration and would be statistically inappropriate as a control [note that the highest values are almost identical between the control (λ=750 nm) and the mutant (λ=720 nm)]. The duration was significantly and consistently shorter at λ≥750 nm in the *lws^–^* mutants. It should be noted that, compared with λ=850 nm (the lowest value), the duration was significantly and consistently longer at λ≤750 nm in the mutant fish (see the Results section). (C) Distance swam by the fish when following the rotating stripes (distance of the OMR). When fish swam in the direction opposite to the stripe rotation, the distance was added as a negative value. The distance decreased significantly at λ≥830 nm and λ≥740 nm for the control and mutant fish, respectively. The decrease observed for the control fish at λ=840 nm was not significant because of the large variance observed. *P<0.05, **P<0.05.

First, we measured the time until the correlation coefficient reached a value of 0.9 after the start/switching of the stripe rotation (Fig. 3A). This parameter was the criterion that we previously adopted to detect the OMR manually; i.e. we judged the OMR as being positive when fish started following the stripes within 10 s of the start/switching of the rotation (Homma et al., 2017). If the correlation coefficient reached the value of 0.9 within 1 s, we regarded it as an accidental correlation and ignored the datum. In addition, if the correlation coefficient never reached the value of 0.9 within 30 s, we regarded the datum to be 31 s (i.e. underestimation of the time). At λ=720 nm, the correlation coefficient (average of three rotations) reached the value of 0.9 within 10 s in all 11 fish, except for one mutant in which the correlation coefficient reached that value in 10.1 s. At λ=750 nm, the mutant animals exhibited a significant delay (23.6±2.7 s; mean±s.e.m.; *P*<0.05, Steel's test), whereas all control fish still responded within 10 s (6.1±0.9 s). This suggests that, at λ=750 nm, the photosensitivity was reduced only in the *lws^–^* mutants. The situation was the same at λ=800 nm; i.e. the mutant animals showed a significant delay (20.3±1.8 s), whereas the control fish still responded quickly (9.9±2.0 s). At λ=850 nm, however, the control animals showed a significant delay (16.5±5.0 s; *P*<0.05), similar to that observed for the mutant fish (23.7±2.8 s). This indicates that both the control and mutant fish could not efficiently perceive light at this wavelength.

Second, we measured the time during which the correlation coefficient was kept at a value of 0.9 or higher (Fig. 3B). We adopted this parameter because the correlation coefficient was stable at a value of nearly 1.0 when the typical OMR was displayed (Fig. 2B), whereas it only accidentally reached the value of 0.9 when fish did not show an OMR (Fig. 2D). Similar to that described above for the first parameter, we ignored the first second in each rotation; therefore, the total observation time was 87 s (29 s/rotation×3 rotations) per test. In the control, the time during which the correlation coefficient was kept at a value of 0.9 or higher (i.e. duration of the OMR) was about ≥50% of the total observation time at λ=720, 750, and 800 nm, but dropped to 11.3%±5.1% at λ=850 nm, although the difference was not significant (see the figure legend). In regards to the mutant fish, in which duration of the OMR was 69.5%±3.5% of the total observation time at λ=720 nm, this percentage was significantly decreased to 20.4%±4.0% at λ=750 nm and at all longer wavelengths, to finally drop to <1% at λ=850 nm (*P*<0.05). Thus, this parameter was also effective in demonstrating the difference in red-light sensitivity between the control and mutant fish. In addition, this result indicated that the *lws^–^* mutants might perceive light at λ=750 nm, because the ratio (20.4%±4.0%) was significantly higher than that recorded at λ=850 nm (0.9%±0.5%; *P*<0.05).

Third, we calculated the total distance moved with the stripe rotation (Fig. 3C). Because the speed of stripe rotation was 6 rpm, fish should complete nine rounds of movement in the 90 s O–O test if they followed the rotating stripes exactly. Interestingly, the migration distances recorded at λ=720 nm were 8.9±1.3 and 11.3±0.8 rounds for the control and mutant animals, respectively, indicating that the fish tended to swim faster than the rotating stripes (note that the direction of rotation was switched twice during the test). At λ=850 nm, the distance was significantly decreased (*P*<0.05) to 1.3±1.3 and 0.3±0.5 rounds in the control and mutant animals, respectively, demonstrating that both types of fish did swim (according to the angular velocity; data not shown), but not necessarily as a result of an OMR. Significant decreases were also detected at λ=750 and 800 nm in the mutant fish (–1.1±0.6 and 0.9±0.9 rounds, respectively), whereas the control fish still migrated for 8.9±1.3 and 7.4±1.5 rounds at these wavelengths, respectively. These results demonstrated clearly that, at λ≥750 nm, photosensitivity was severely reduced in the *lws^–^* mutants.

## DISCUSSION

### The OMR as an index to investigate spectral sensitivity in small animals

In this study, we developed a low-cost and semi-automatic method for quantifying the OMR. It required simply the x–y coordinates of a test animal, which were collected efficiently using a free software, the UMATracker program (Table 1). Based on these coordinates, three parameters that consistently supported a significant difference in spectral sensitivity between the control and *lws^–^* medaka (Fig. 3) were calculated using generic spreadsheet software (Excel). It is worth emphasizing that we never stopped or repeated the O–O test (i.e. we analyzed all x–y coordinates as they were), even when a lack of interest in the rotating stripes was suspected (e.g. Fig. 2F). Nevertheless, the three parameters successfully demonstrated a progressive attenuation of the OMR at longer wavelengths in both the control and mutant fish (Fig. 3). Thus, this quantitative O–O test would be of further usefulness to investigate behavioral photosensitivity, even for researchers with little knowledge of the OMR or the behavioral characteristics of test animals.

The optokinetic response [OKR; saccadic movements of the eyes to pursue moving objects (Tabata et al., 2010)] or electroretinography (ERG; electrophysiological response of the retina) are other methods that could be used to investigate spectral sensitivity; in fact, they have been used to study other species (Brockerhoff et al., 1995; Dowling and Sidman, 1962; Strother and Casella, 1972). The advantages of these methods are that the response (movements or action potential in the eyes) is simple and can be easily analyzed. Another merit would be that these responses always occur whenever stimuli are present; the responses are more innate and hardly affected by the animal's inclination to swim or move, unlike the OMR. However, these methods require the stabilization of the test animal, which causes physical and psychological stresses, and it is unknown how these affect the measurements of visual performance.

Conversely, the OMR is less stressful for animals, as the animals move freely during the test; however, this complicates the interpretation of the behavior (e.g. Fig. 2G,H). Although our O–O test could be optimized further (e.g. the diameter of the tank, the speed/duration/number of stripe rotations, video recording at the edge of the tank, binarizing filters, and the parameters), the present study may provide a more stress-free environment for the animals while affording a sensitive (see below) method to analyze the quantitative changes in spectral sensitivity. In addition to the red-colorblind *lws^–^* medaka, we are currently establishing green-, blue-, or violet-colorblind medaka by introducing mutations into the *RH2*, *SWS2*, or *SWS1* genes, respectively (unpublished). Although the *rh2^–^* or *sws2^–^* mutants with intact SWS1 and LWS vision will likely perceive and exhibit an OMR under green or blue light, the present method might be helpful to detect decreased photosensitivity at these wavelengths.

### Spectral sensitivity of medaka

We reported previously that the longest sensible wavelength was 830 nm in medaka (Homma et al., 2017), which was much longer than the λ_max_ of LWSa and LWSb [561 and 562 nm, respectively; (Matsumoto et al., 2006)]. The present study detected significant differences in photosensitivity at λ≥830 nm, but not at λ≤820 nm (Fig. 3), showing a surprising concordance between the manual and semi-automatic assessments. However, we suspect that medaka may perceive longer wavelengths (e.g. 840 nm), considering the progressive increase/decrease in the three parameters toward λ=850 nm (Fig. 3). The same was true for the *lws^–^* mutants, as shown by the results reported previously by our group (the longest sensible wavelength was 730 nm); the present study did not detect significant differences up to 730 nm, but all parameters continued to increase/decrease toward λ=800 nm (or 850 nm; Fig. 3).

Using the OMR, OKR, and ERG or other methods, the spectral sensitivity of various fish has been measured. Although many of these studies used λ=600–700 nm as the longest light wavelength [e.g. Enright et al. (2015); Harrington et al. (2015); Horodysky et al. (2013); Kalinoski et al. (2014); Sakai et al. (2016); Shao et al. (2014)], some studies focused on the near-infrared (NIR) region and successfully demonstrated NIR sensitivity in carp, cichlids, guppy, platy, and zebrafish [see Shcherbakov et al. (2013) and the references therein]. Based on the ERG, sensitivity was low at wavelengths longer than 700 nm, whereas a phototactic assay exhibited sensitivity at over 900 nm (Shcherbakov et al., 2013). Thus, it is obvious that different methods have different spectral sensitivities and we do not exclude the possibility that medaka perceives NIR light at wavelengths longer than 850 nm. However, whether such slight (which is not manually detectable) sensitivity is essential or advantageous for the animals in nature (e.g. for stabilizing positions, finding prey, or escaping from predators) or is merely a by-product of spectral tuning of LWSs at shorter wavelengths remains to be demonstrated empirically.

In summary, we developed a new method to quantitate the OMR that is objective, semi-automatic, inexpensive, and more sensitive than manual observation. Even when using minimal sample sizes (*n*=5 or 6), the results clearly demonstrated significant differences between medaka with normal and red-colorblind vision. This method might be applied to other fish and land animals, which would help complete various OMR-based studies.

## MATERIALS AND METHODS

### Animals and care

All experiments described herein were conducted in accordance with the Code of Ethics of the World Medical Association (Declaration of Helsinki). We used four medaka (*O. latipes*) strains in this study; *color interfere* (*ci*) and Actb-SLα:GFP with or without the double-frameshift mutations (*lws^+2a+5b^*) on the paralogous *LWSa* and *LWSb* genes (Homma et al., 2017). In this article we refer to the fish with or without mutations as mutant or control fish, respectively. All fish were hatched and bred in our laboratory, where tanks were maintained under a 14/10 h light/dark rhythm using standard fluorescent lamps. Only fully matured fish were used, because cone-cell density and visual acuity increase gradually during the larval stages (Ohki and Aoki, 1985).

### OMR test using the OLS; the O–O test

**Fig. 4.**
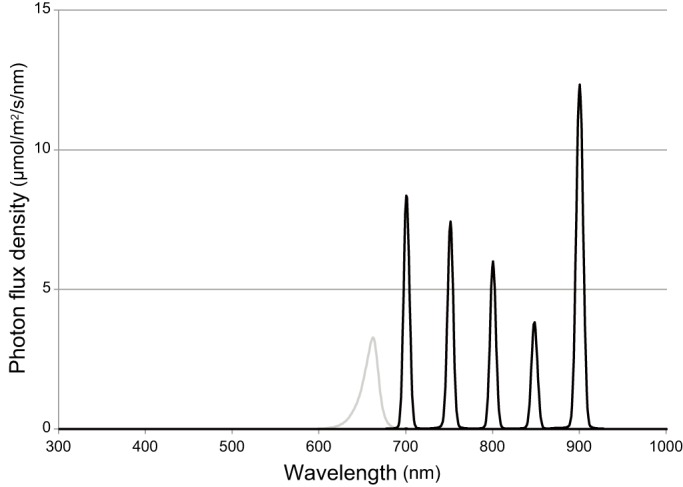
**Spectra of monochromatic light from the OLS.** Spectra were measured separately at λ=700, 750, 800, 850, and 900 nm using a spectroradiometer (S-2440C; Soma Optics, Tokyo, Japan), but were illustrated together in this figure as black lines. The difference in intensity (see Table 2) reflected the spectrum of the light source, which was a xenon arc lamp (Watanabe et al., 1982). The gray line indicates the spectrum of light from red LED lamps (VBL-S300-R; Valore, Kyoto, Japan), with a peak wavelength at 660 nm.

We adopted a procedure described elsewhere to video record fish behavior during the O–O test (Homma et al., 2017). Briefly, we used the electronic apparatus to rotate black and white stripes (2 cm wide) around a cylindrical glass tank with a diameter of 18.5 cm. The speed of the rotation was ∼6 rpm (36°/s) and the direction could be switched arbitrarily. The tank was irradiated from the top using parallel monochromatic light sources generated by the OLS at the National Institute for Basic Biology [(Watanabe et al., 1982); see Fig. 4 and Table 2 for the photon densities measured in the apparatus], and fish in the tank were silhouetted on a white plastic paper under the tank. We recorded these silhouettes using an A10FHDIR video camera (Kenko). The infrared (IR) light that irradiated from the camera in the IR-recording mode was shielded by covering the IR bulb with aluminum foil. By closing the shutters of the OLS, we prevented light with a wavelength shorter than the tested light from coming into the OLS room.

**Table 2.**
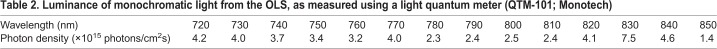
**Luminance of monochromatic light from the OLS, as measured using a light quantum meter (QTM-101; Monotech)**

We placed one fish that had been light adapted over 5 min under fluorescent lamps of about 5×10^14^ photons/cm^2^s in the cylindrical tank. At the center of the tank, we placed a water-filled 50 ml centrifuge tube, to prevent shortcut swimming by the fish during the OMR (the shortcut complicates the analyses of angular velocity and correlation coefficient). Subsequently, we turned the OLS on, turned the ceiling lights off, started the video recording, waited for 30 s without rotating the stripes (for acclimation), then rotated the stripes in a clockwise, counterclockwise, and clockwise direction for 30 s each. The rotation and recording were then stopped, the ceiling lights were turned on, and the OLS was turned off. The fish were light adapted again before being subjected to the next test, which was performed at a different wavelength. To prevent the potential adaptation to invisible light [i.e. dark adaptation, which provokes rod vision; (Homma et al., 2017)], we always shifted the wavelength from a shorter to longer one during the series of O–O tests.

### Tracking and data analyses

The recorded behavior was changed into a series of numeric x–y coordinates (one coordinate in a frame) using the UMATracker program, which is freely available on the internet (http://ymnk13.github.io/UMATracker/). We first trimmed the movie, if frames at its beginning or end captured fluctuating luminance or trembling motions of the apparatus. Subsequently, we binarized the movie using appropriate filters produced by the UMATracker, tracked the position of medaka using the ‘K-means (w/o tracking)’ algorithm, and exported the coordinates as a text file. The coordinate of the center of the cylindrical tank and the frames at which the rotation was started/switched/stopped were determined manually.

Using the Excel software (Microsoft), the x–y coordinates were transformed into angular coordinates, to calculate the angular velocity and correlation coefficient. These two parameters were calculated using two contiguous frames, but were averaged every 60 frames (i.e. 1 s), because the x–y coordinates often fluctuated to a minute degree, even when the fish were not moving. The correlation coefficient was calculated as the cosine between the direction of swimming and the tangential direction of the rotating stripes at the coordinate. If a coordinate was missing from a frame (because of a tracking failure), we ignored the neighboring frames and did not calculate the angular velocity or correlation coefficient. If coordinates were missing in more than 30 frames in 60 contiguous frames, we did not calculate the averaged angular velocity or correlation coefficient, and treated them as missing data.

Using the angular velocity and correlation coefficient, we calculated three parameters to quantify the OMR. To compare the values among wavelengths per strain, we applied Steel's test between the wavelength of 720 nm and other wavelengths.

## Supplementary Material

First Person interview
