# Supplementary material for: A semi-automatic and quantitative method to evaluate behavioral photosensitivity in animals based on the optomotor response (OMR)
Source: Biol Open. 2018 Jun 15;7(6):bio033175. doi: 10.1242/bio.033175 (PMC6031347; doi:10.1242/bio.033175)
Supplement: First Person interview [file biolopen-7-033175-s1.pdf]

FIRST PERSON

First person – Megumi Matsuo

First Person is a series of interviews with the first authors of a selection of papers published in Biology Open, helping early-career researchers promote themselves alongside their papers. Megumi Matsuo is first author on 'A semi-automatic and quantitative method to evaluate behavioral photosensitivity in animals based on the optomotor response (OMR)', published in BiO. Megumi is a teaching associate and researcher in the lab of Shoji Fukamachi at Japan Women's University, Tokyo, Japan, using a combination of *in vivo*, *in vitro* and *in silico* analyses to draw out answers from chaotic subject matter.

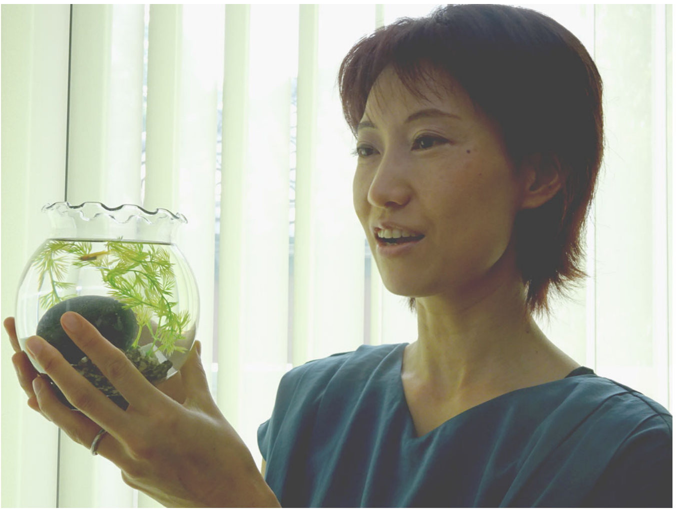

Megumi Matsuo with medaka.

What is your scientific background and the general focus of your lab?

I was a fellow of the Japan Society for the Promotion of Science and completed my undergrad studies with a biology degree in evolutionary biology of the immune system at the University of Tokyo. After my undergrad work, I joined a project of the Organization for Drug ADR Relief R&D Promotion and Product Review in Japan. As a PhD fellow, my primary work was correlation analysis to unlock the causes of a number of human hereditary diseases. I left the research field for several years, and now am involved in a project to understand the influence of body color on evolutionary processes in the lab of Dr Shoji Fukamachi. We use medaka, tiny cute freshwater bony fish, as a model. Many medaka inbred lines with identical backgrounds and many mutants are available, and medaka is transparent during the embryonic stage, so this bony fish is an ideal animal for scientific research.

How would you explain the main findings of your paper to non-scientific family and friends?

Sometimes we are not sure whether an animal can see an object or not. Thus, we developed a semi-automatic system to easily discern whether an animal can see or not, using the optomotor response (OMR). The OMR is a visually induced locomotor behavior of animals pursuing a moving repetitive stimulus pattern, which can be observed in water and land animals. This ability allows animals to perform synchronized and coordinated movements, such as flocking and schooling behavior. Our semi-automatic system was established using medaka, small freshwater fish. Our method requires no special skills or knowledge about fish and is a really animal-friendly technique. This low-cost, quantitative and semi-automatic method widens opportunities for researchers to unveil behavioral photosensitivity in animals of interest.

What changes do you think could improve the professional lives of early-career scientists?

Grants and wide knowledge. After completing my PhD, I needed to devote myself to caring for my children, as my eldest girl suffered from severe asthma. I was never pessimistic and loved caring for her, but if I could have had support from the care services for sick and recovering children, my life would have been very different. After several years passed, the time came for me to return to the research field, but age limits on opportunities for early-career researchers had become a limiting factor for me. Of course, age limits are beneficial to young researchers, but multifarious financial support is needed, for example, to provide opportunities to scientists who have spent time away from research for personal reasons.

During the time I spent raising and caring for my three girls, I had a chance to move to the USA, but not as a scientist. It was such an inspiring experience! I had discussions with many people from all over the world, with different backgrounds. Sometimes we talked about political matters, or ways to conserve the environment and save the earth. I believe that wide knowledge and communications are so precious, and similar experiences will always inspire early-career scientists.

"[…] my dream is to help others like me who are re-starting their careers after a break."

What's next for you?

Megumi Matsuo is back on the starting line. I want to absorb as much current knowledge as I can. Previously, I learned both experimental work (so called 'wet work') and computational analysis ('dry work'); my next step is to become an expert in both fields. Employing both techniques, I want to challenge a new field. What new field? Honestly, I don't have anything specific in mind at present, but I am looking out for it. Once I find this new field to challenge and successfully lead a project, my dream is to help others like me who are re-starting their careers after a break. As a mother, wife and now as a scientist, my next step is kicking off!

Megumi Matsuo's contact details: Department of Chemical and Biological Sciences, Japan Women's University, Mejirodai 2-8-1, Bunkyo-ku, Tokyo 112-8681, Japan.
E-mail: mmatsuo@fc.jwu.ac.jp

Reference

Matsuo, M., Ando, Y., Kamei, Y. and Fukamachi, S. (2018). A semi-automatic and quantitative method to evaluate behavioral photosensitivity in animals based on the optomotor response (OMR). *Biol. Open* **7**: bio033175.
